# Supplementary figures and images for: Randomizing Genome-Scale Metabolic Networks
Source: PLoS One. 2011 Jul 14;6(7):e22295. doi: 10.1371/journal.pone.0022295 (PMC3136524; doi:10.1371/journal.pone.0022295)

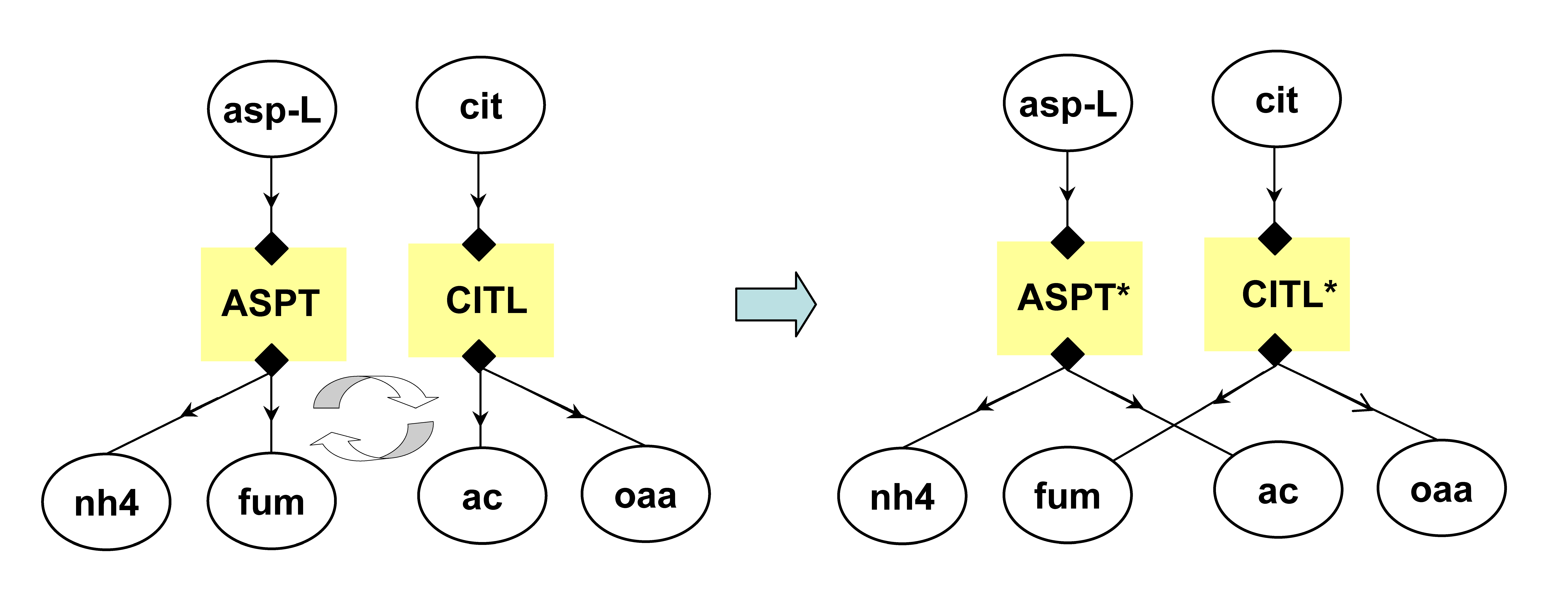

Supplement: Figure S1 — Edge exchange randomization is biochemically meaningless. The commonly used edge exchange or link permutation procedure for randomizing biological networks is inappropriate for metabolic networks as the method generates fictitious reactions violating balance of mass, charge and atomic elements. Here, starting with two reactions (ASPT: asp-L → fum + nh4; CITL: cit → oaa + ac), we perform an edge exchange associated to metabolites fum and ac that generates two new hypothetical reactions (ASPT*: asp-L → ac + nh4; CITL*: cit → oaa + fum) that violate balance of mass and atomic elements. Note that ac has 2 carbon atoms and fum has 4 carbon atoms. (TIF) [file pone.0022295.s001.tif]

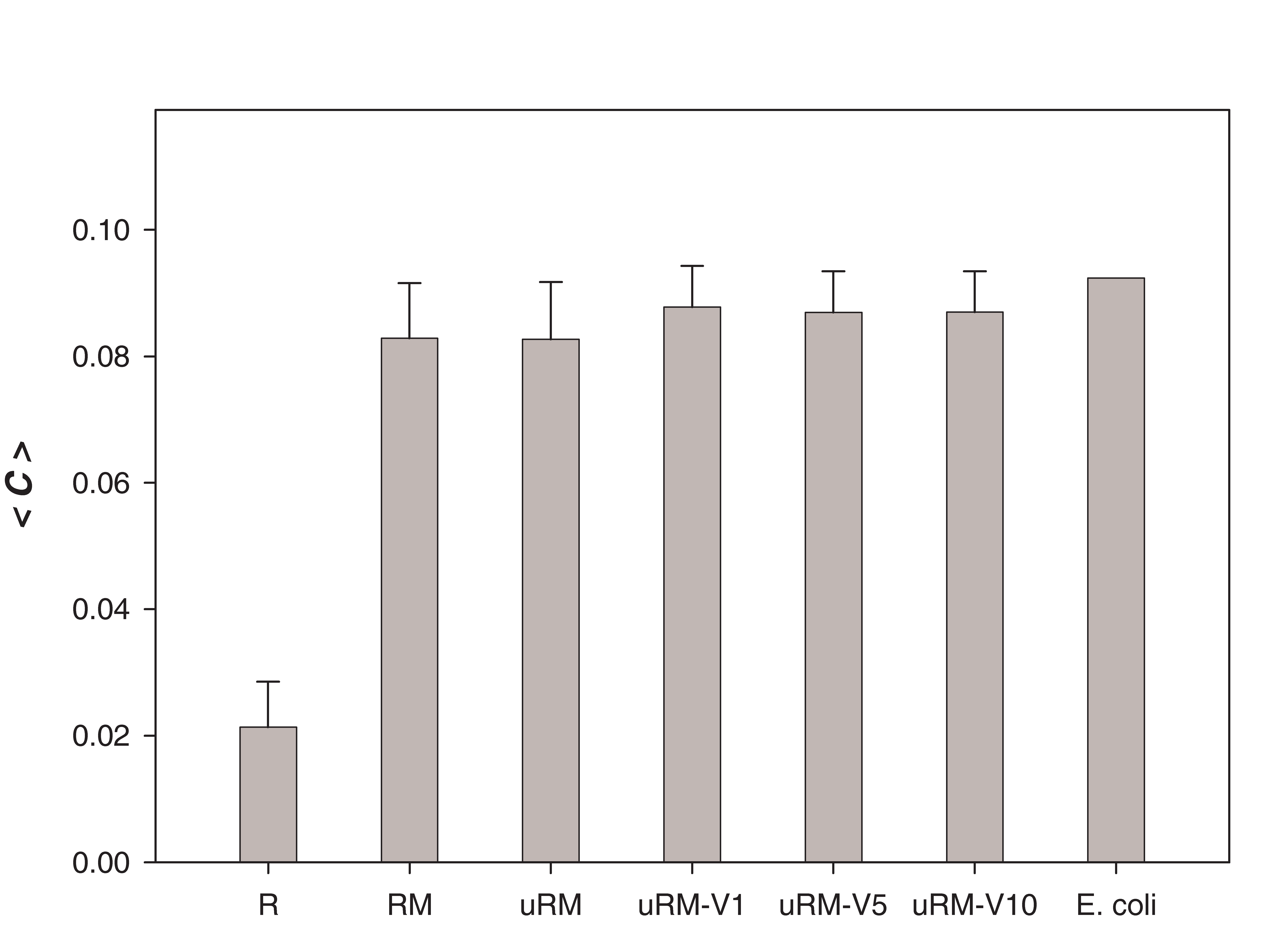

Supplement: Figure S4 — Clustering coefficient C of the metabolic networks in the different ensembles. Different bars from left to right correspond to network ensembles incorporating an increasing number of constraints and the last bar corresponds to the E. coli metabolic network. The standard deviation is also displayed for each ensemble. (TIF) [file pone.0022295.s004.tif]

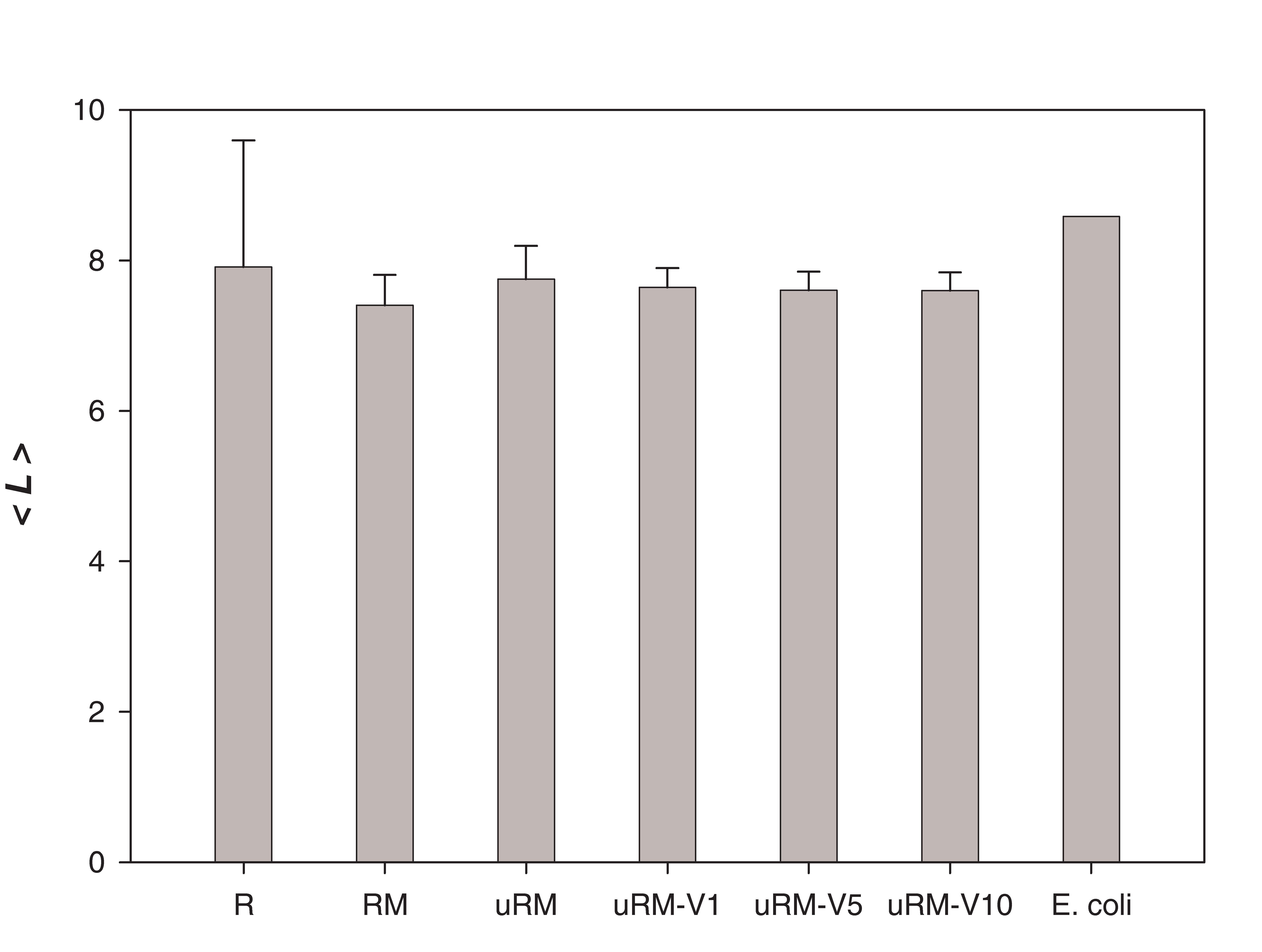

Supplement: Figure S5 — Average path length < L > of the metabolic networks in the different ensembles. Different bars from left to right correspond to network ensembles incorporating an increasing number of constraints and the last bar corresponds to the E. coli metabolic network. The standard deviation is also displayed for each ensemble. (TIF) [file pone.0022295.s005.tif]

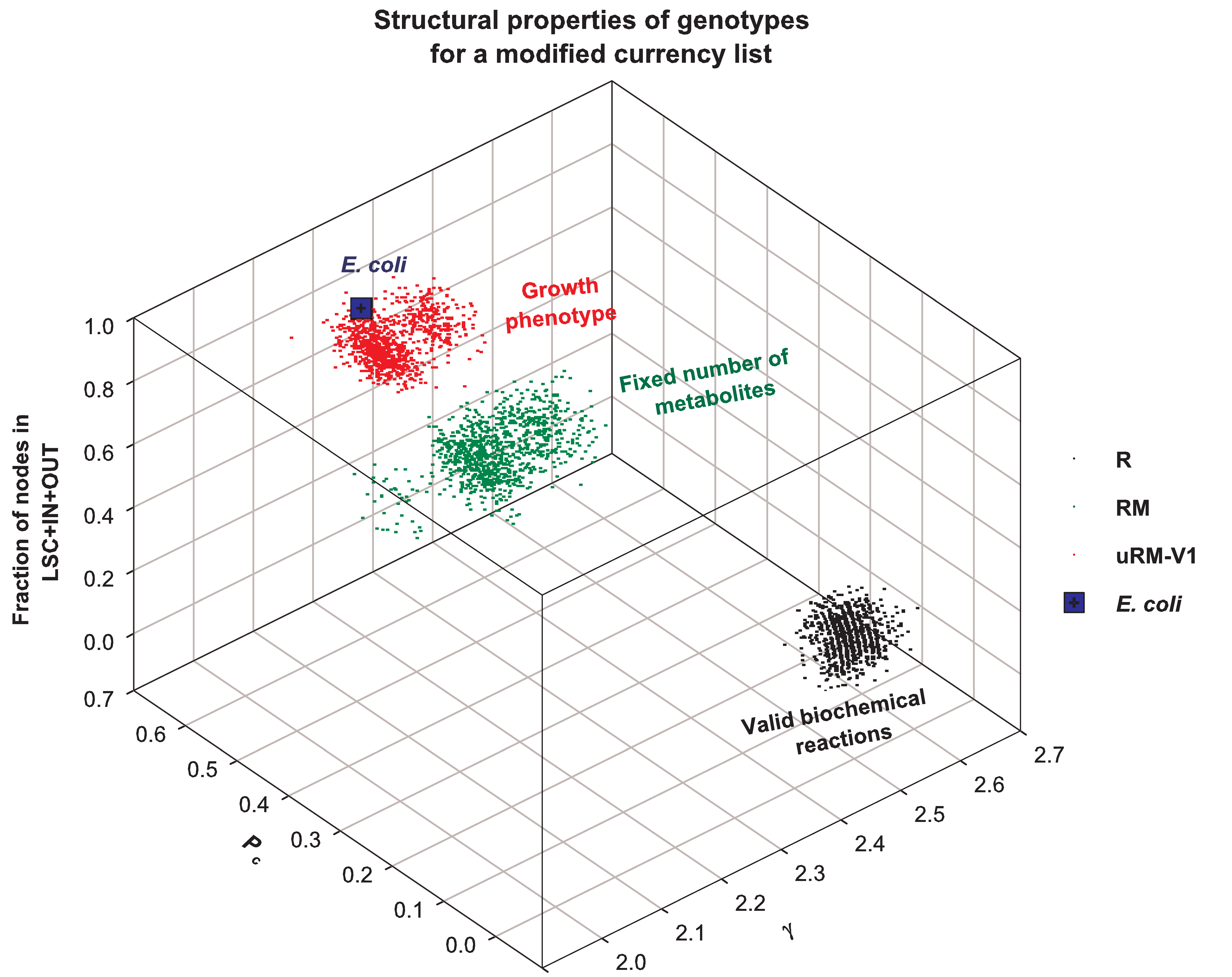

Supplement: Figure S6 — Statistical properties of randomized networks in different ensembles and the E. coli metabolic network using a modified currency list. The three axes are associated with graph characteristics of the networks and are same as in Figure 6. Each cloud represents 1000 randomized networks in the ensemble considered. In order to compute graph characteristics of randomized networks shown in this figure, we have constructed the metabolite-metabolite graph corresponding to each randomized network using a currency list modified from that listed in Table S1. The modified currency list was generated as follows. We first ranked metabolites in the currency list (given in Table S1) based on metabolite degree in the complete reaction database. The lowest degree metabolite in the currency list was designated rank 1. The 20 metabolites of smallest rank in this ranked currency list were then eliminated to generate the modified currency list used for computing graph characteristics shown in this figure. By comparing this figure with its analog (Figure 6), one sees that our conclusions are the same for the two definitions of currency metabolites. (TIF) [file pone.0022295.s006.tif]
